# Artificial intelligence in medicine: a position paper by the Italian Society of Internal Medicine

**DOI:** 10.1007/s11739-025-04146-4

**Published:** 2025-12-19

**Authors:** Clara Balsano, Federico Cabitza, Sebastiano Cicco, Marco Gori, Donato Malerba, Marco Montagna, Roberto Tarquini, Angelo Vacca

**Affiliations:** 1 Geriatric Unit, School of Emergency-Urgency Medicine, Department of Life, Health and Environmental Sciences-MESVA, University of L’Aquila, L’Aquila, Italy; 2 Department of Informatics, Systems and Communication, University of Milano-Bicocca, Milan, Italy; 3 IRCCS Ospedale Galeazzi Sant’Ambrogio, Milan, Italy; 4 Department of Precision and Regenerative Medicine and Ionian Area (DiMePRe-J), Unit of Internal Medicine “Guido Baccelli”, University of Bari Aldo Moro, Bari, Italy; 5 Department of Precision and Regenerative Medicine and Ionian Area (DiMePRe-J), Telemedicine Research Center, University of Bari Aldo Moro, Bari, Italy; 6 Department of Information Engineering and Mathematics, University of Siena, Siena, Italy; 7 Department of Informatics, University of Bari Aldo Moro, Bari, Italy; 8 School of Medicine, Vita-Salute San Raffaele University, Milan, Italy; 9 SOC Medicina Interna, USL Toscana Centro, Empoli, Italy

**Keywords:** Advocacy, Checklists, Explainable AI, Key opinion leaders, Machine learning, Recommendations

## Abstract

Artificial Intelligence (AI) represents an innovative technological support for clinical practice. The Italian Society of Internal Medicine (SIMI) emphasizes the need for clear guidance on the use of AI in medicine, recognizing that knowledge in this field is continuously evolving. This position paper presents a comprehensive vision for the responsible integration of AI into clinical practice. AI should serve as a support tool—not a replacement—for clinicians. It has the potential to improve diagnostic accuracy, reduce administrative workload, and strengthen the physician–patient relationship. In the light of these characteristics, SIMI advocates for transparency, data privacy, equity, and sustainability in the development and implementation of AI systems. SIMI also highlights several ethical, legal, and methodological challenges that must be addressed, including algorithmic bias, environmental impact, and disparities in access. Ultimately, SIMI envisions a future in which AI augments human expertise, enabling more efficient, personalized, and compassionate care. SIMI calls for active clinician participation in the co-design and validation of AI tools to ensure alignment with real-world clinical needs. Key recommendations include the preferential use of certified AI systems, the integration of AI education into medical training, and continuous monitoring after deployment.

## Premise

It is unanimously recognized that the whole healthcare sector, both at its systemic (national/international healthcare systems) and individual level (practitioners, patients and caregivers), is currently facing great challenges. Aging populations, growing patient complexity, rising care delivery costs, staff and resource shortages, only to mention a few, call for a substantial revision of healthcare at all levels, if health is to remain a universal human right. Such revision would contemplate the adoption of new technologies, among which Artificial Intelligence (AI) is the one holding the greatest potential for beneficial impact. AI is expected to enhance diagnostic, monitoring, and prognostic capabilities, advance precision medicine, reduce costs and workloads across both clinical and administrative domains, accelerate the discovery of new therapies, and generate synthetic data for clinical trials, just to name a few.

Recognizing this potential, but also the critical possible risks linked to the application of AI to healthcare, the Italian Society of Internal Medicine (SIMI) founded a working group to explore and discuss the topic and foster research on it.

This position paper marks the beginning of the working group, expresses the view of the Society on such a hot and debated theme and is a call for action and continuous education to all Internal Medicine specialists. Therefore, the manuscript begins by sharing introductory concepts to AI and definitions of its components. We then list ten recommendations for its particular use in Internal Medicine. Along the manuscript, we highlight, on one hand, promises and game-changing potential of AI and, on the other hand, its pitfalls and associated risks.

Box 1 – Definitions: **Artificial Intelligence:** A suite of computational techniques that enable machines to perform complex intellectual tasks traditionally requiring human cognition. The term now encompasses both the operational systems that execute these cognitive functions and the interdisciplinary field of study devoted to their development, validation, and impact assessment.

**Big data:** Extremely large, heterogeneous and continuously updated datasets (such as health data collected from devices, electronic health records, images, etc.). Analysing such data requires advanced tools to extract useful information for medical practice.

**Deep Learning:** A subset of machine learning that uses artificial neural networks with many layers ("layers") to analyse large amounts of data. It is particularly effective in tasks such as classifying complex radiological images or clinical signals.

**Explainable AI:** Techniques that enhance the interpretability of complex AI systems, especially by clarifying “why” a certain advice or recommendation was given.

**Generative AI:** Systems that generate new content in the form of text, images, videos or sounds. In medicine, they can synthesize clinical information, produce explanatory images, or create training simulations.

**Large Language Models:** Systems that process natural language (such as written or spoken text), learning from huge amounts of content. They could be used, for example, to support reporting or clinical decision making.

**Machine Learning:** A subset of AI techniques and methods that enable computational systems to derive knowledge from data through pattern recognition and statistical inference, rather than through explicit programming. They allow computers to build predictive and classificatory models by identifying meaningful patterns and correlations within large datasets.

## Introductory concepts

**Fig. 1:**
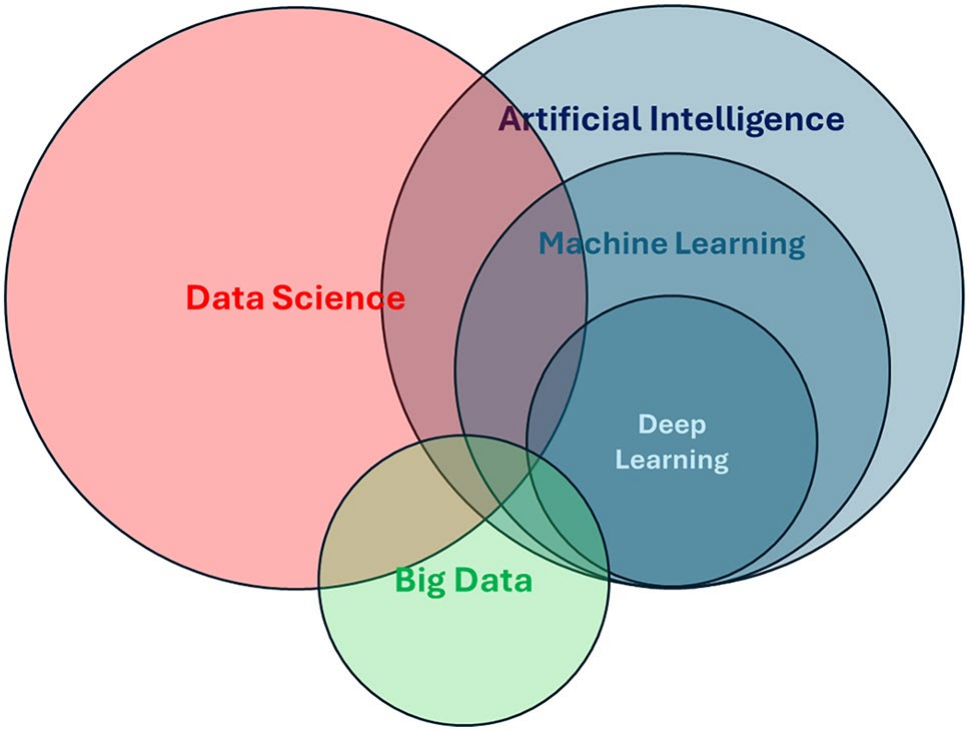
Venn diagram depicting the hierarchical disposition and relative superimposition of key concepts examined in the present manuscript. Artificial Intelligence (AI) is a broad field of computer science concerned with the development of systems capable of performing tasks that typically require human intelligence. These tasks include reasoning, perception, decision-making, learning, and natural language understanding. AI systems may operate in both virtual and physical environments and are designed to act autonomously, analyzing their surroundings and making informed decisions to achieve specific goals. Machine Learning (ML), a core subfield of AI, focuses on algorithms that learn models from data and improve their performance over time without being explicitly programmed for each specific task. ML enables systems to generalize from examples and adapt to novel inputs, thus supporting a wide range of predictive and decision-making applications. Deep Learning is a specialized area within ML that employs deep artificial neural networks, typically composed of many layers, to model complex relationships in data. These architectures have proven especially effective for handling high-dimensional data such as images, speech, and natural language, and have powered recent advances in AI, including the development of Large Language Models (LLMs). Data Science is an interdisciplinary domain focused on extracting meaningful insights and knowledge from structured and unstructured data. It integrates methods from statistics, computer science, and specific application domains to analyze data, uncover patterns, and support decision-making. Whereas AI focuses on the autonomous decision-making of systems acting within their environment, Data Science is chiefly oriented toward supporting human insight through the exploration, analysis, interpretation, and visualization of data. Big Data refers to datasets that are too large, complex, or fast-changing to be effectively managed with traditional data processing tools. It is typically defined by the “3 Vs”: Volume, Variety, and Velocity. Big Data serves as a crucial enabler for both Data Science and AI, providing the scale and richness of information needed to train sophisticated models and uncover fine-grained patterns in large-scale systems

**Fig. 2:**
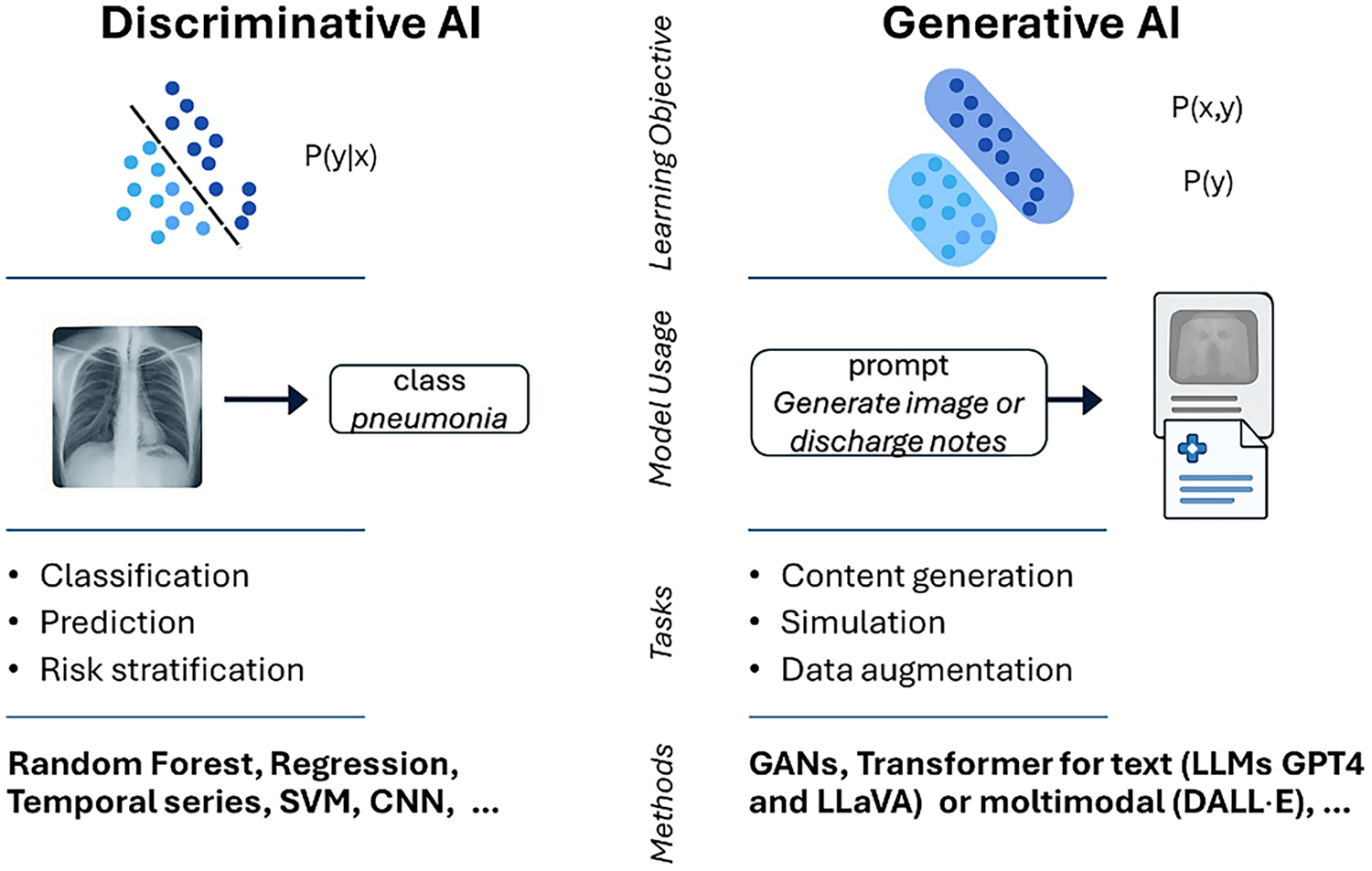
Conceptual and probabilistic distinctions between discriminative and generative approaches in Artificial Intelligence (AI). Left: Discriminative AI focuses on learning the conditional probability distribution P(y∣x), that is, the probability of a label y (e.g., a disease diagnosis) given input data x (e.g., a chest X-ray). Discriminative models are trained to distinguish between classes and are optimized for tasks such as classification, prediction, and risk stratification. Common examples include Random Forests, regression models, Support Vector Machines (SVMs), Convolutional Neural Networks (CNNs), and ARIMA. These techniques are widely employed in Clinical Decision Support Systems (CDSS) and automated diagnostic tools. Right: Generative AI, on the other hand, focuses on learning the joint or marginal distributions of data, P(x, y) or P(x), i.e., the probability of data-label pairs or the data itself. This enables the generation of new, realistic data samples that reflect the underlying structure of the training distribution. In the illustration, a prompt leads to the generation of medical documentation, symbolizing tasks such as content creation, simulation, and data augmentation. Generative models are trained to learn these distributions and include approaches such as Generative Adversarial Networks (GANs), Variational Autoencoders (VAEs), and transformer-based models. Foundation models typically emerge from the latter category. They are pre-trained on massive corpora of text (e.g., GPT-4, LLaVA) and increasingly on multimodal data (e.g., DALL·E), and can then be fine-tuned or prompted for specific downstream applications. These models exemplify the generative paradigm by responding flexibly to natural language prompts and integrating different modalities (e.g., vision and language) to support complex tasks such as radiology report generation, synthetic medical image creation for training purposes, or the simulation of rare clinical scenarios

Before delving into the details of AI applications to healthcare, with a particular focus on Internal Medicine, we provide a short introduction to AI and its vocabulary that should serve as a common ground for the subsequent sections of the paper (Box 1, Fig. 1) [1]. AI appears as a large field that comprises multiple techniques, partially overlapping with the field of Data Science and often leveraging big data, especially in more advanced applications (i.e., Deep Learning). We also describe the fundamental distinctions between discriminative and generative approaches in AI (Fig. 2). They respectively allow for either classification/prediction tasks or content generation/data simulation tasks. These concepts represent the core body of knowledge that we consider essential for all medical professionals to possess, irrespectively of their occupation or specialization.

The need of the Internal Medicine position paper to be presented here.

AI offers multiple promising opportunities in clinical settings. Its ability to process huge amounts of data and generate human-like responses can support a number of healthcare applications—from image analysis [2–4] to clinical evaluation [5, 6]. AI is emerging as a valuable tool in medicine [7–9], particularly for its role in supporting clinical decision-making [10, 11]. Evidence suggests that AI may even assist in communicating with patients in specific contexts [12–16]. However, alongside this enthusiasm, several critical questions have arisen. As an example, recent studies have explored the capabilities of Large Language Models (LLMs) in clinical conversational reasoning, history-taking, and diagnostic accuracy [17]. However, while LLMs show potential, these studies have not demonstrated yet superiority over traditional clinical judgment [18]. Thus, to date, LLMs may still serve only as useful support tool in practice.

Moreover, both patients [19] and physicians [20] have expressed resistance to adopting AI, particularly when it is perceived as a replacement for human clinicians. Ethical and technical concerns remain significant barriers. Other limitations include the quality and completeness of data used to train AI models [21], which can lead to suboptimal performance [22]. These challenges have raised important ethical considerations regarding the use of AI in healthcare and scientific writing [23].

On the technical side, literature presents diverse and sometimes contrasting positions on the role of AI in medicine. Some works emphasize the superiority of AI systems in specific tasks, such as diagnostic image interpretation or pattern recognition, showing performance levels comparable or even superior to human experts in narrow domains [2, 17]. Others, however, stress that these results are often context-dependent, obtained in controlled research settings, and difficult to generalize to the complexity of real-world clinical practice [18, 24]. A further debate concerns the data-centric vs. model-centric approaches: while some scholars argue that progress will mainly come from increasingly sophisticated algorithms, others point out that the real bottleneck lies in the quality of training data [25]. There are also different technical views on the integration of AI with clinical workflows: some propose full automation for repetitive or low-risk tasks, others insist that AI should remain strictly a decision support system, leaving final responsibility to human clinicians [26, 27]. This reflects a broader debate on whether AI will eventually replace doctors [24, 28, 29] or instead serve as a powerful ally in clinical practice [26, 27, 30].

Finally, a key pedagogical challenge is ensuring that future physicians learn to critically appraise and validate AI outputs rather than accepting them uncritically. In fact, beyond direct clinical applications, AI is being tested in medical education and training of clinicians. It can generate simulated clinical cases, flashcards, and quizzes to support students’ learning [31], as well as simulate doctor–patient conversations or history-taking for communication skills training [32]. Nevertheless, his use remains complementary: to date, it has not shown superiority over traditional clinical reasoning [33].

In this context, the Italian Society of Internal Medicine (SIMI) emphasizes the need for a clear position to better understand the capabilities and limitations of AI. The goal is to harness its technical potential while minimizing risks, ensuring its responsible integration into daily clinical practice, research, and education.

## Recommendations and guiding principles

1. **AI technologies in healthcare should serve as supportive tools that aid, rather than replace, the clinical reasoning and decision-making of clinicians and other healthcare professionals.**

**Table:** 

Possible risks	Weaknesses	Potential solutions
Overreliance on AI may erode clinical judgment	AI may be misused for cost-saving, compromising care quality	Promote clinician-led decision-making and emphasize AI as a support tool
Reduced human interaction in care	AI may overlook rare or atypical conditions	Reinforce the clinician–patient relationship and uphold human-centered care

AI holds significant transformative potential for healthcare. By accelerating decision-making processes and streamlining data analysis, AI can help address many of the current limitations within healthcare systems, particularly in settings characterized by workforce shortages and constrained resources.

SIMI strongly supports the future integration of AI in healthcare. However, it is essential to ensure that healthcare remains fundamentally human-centered. Healthcare professionals have a vocation and responsibility to serve and protect human life. Therefore, the clinician–patient relationship must remain the foundation of medical practice. Empathy, holistic care, and attention to the person—rather than merely the disease—must continue to define healthcare systems. Upholding these values necessitates a clinician-led model of care.

Clinicians are uniquely equipped to address the complexity of the whole person, while AI can support them by rapidly analyzing specific data points [4, 8, 34–36]. This collaboration enables clinicians to allocate more time to direct patient care and contributes to a more efficient use of limited resources [8, 34, 37, 38].

SIMI also underscores the potential of AI in reinforcing existing medical knowledge. The COVID-19 pandemic highlighted the vital role of clinical intuition and judgment, particularly in the face of novel diseases. In this context, SIMI envisions AI as a tool that supports—but does not replace—clinical reasoning, within a clinician-centered framework. This approach fosters a collaborative—or even “symbiotic”—relationship, wherein artificial and human intelligence work together to enhance medical decision-making [8, 38].

AI’s ability to process large volumes of data can significantly reduce the time required for differential diagnosis [6, 9, 39, 40]. By identifying relevant patterns, AI can assist clinicians in making more accurate and personalized treatment decisions earlier in the care pathway [41–43]. In addition, AI may help bridge knowledge gaps within specific medical specialties [44–46]. Even in Internal Medicine, where broad expertise is crucial, rare or atypical conditions may be overlooked. AI can aid in detecting such cases, but the final clinical judgment must always remain with the physician.

Finally, SIMI cautions against the misuse of AI in healthcare. While AI can be useful for collecting preliminary information, direct patient interaction with AI should never replace the clinician. A healthcare system driven solely by cost-saving imperatives must not compromise the integrity of the clinician–patient relationship. Preserving this relationship is not only an ethical obligation but also essential to maintaining the quality of care.

As SIMI, we expect this recommendation will help preserve the central role of clinicians in patient care, ensuring that AI augments rather than replaces clinical expertise. This approach is expected to enhance diagnostic accuracy and efficiency, by the use AI as a supporting tool, while maintaining the humanistic values of empathy and holistic care that are fundamental to Internal Medicine. With this structure, healthcare systems can reduce the risk of overreliance on automation, ultimately improving patient safety and sustaining trust in medical decision-making.

2. ** Clinicians should acquire the necessary knowledge and competencies to interact effectively with AI-enhanced healthcare systems. Therefore, there is the need for the integration of AI-related education and training across all levels of medical education and professional development.**

**Table:** 

Possible risks	Weaknesses	Potential solutions
Automation bias due to blind trust in AI outputs	Lack of clinician understanding of AI systems	Introduce AI literacy in medical curricula and continuing education
Misinterpretation of AI due to poor data quality	Limited attention to data quality in model-centric approaches	Promote data-centric AI education focusing on data integrity and bias awareness

It is essential to promote the integration of AI education and training at all levels of medical education and continuing professional development [47–49]. According to the World Health Organization, the full potential of AI to improve healthcare can only be realized if healthcare professionals are equipped to understand, apply, and critically evaluate these technologies within the context of everyday clinical practice [50].

Incorporating AI literacy into both undergraduate and postgraduate medical education is crucial to prepare the healthcare workforce for an evolving, digitally driven health system [51–54]. This requires a rethinking of academic curricula and continuing education programs to include dedicated modules on AI, emphasizing multidisciplinary approaches and scenario-based clinical learning. Consistent with a data-centric AI approach (Box 2), training should also equip clinicians with the skills to assess how data quality, bias, and completeness directly affect the reliability and safety of AI systems [55].

The training of healthcare professionals in the use of innovative AI technologies could also be supported by third-sector organizations already engaged in educational activities [1]. These actors should operate in accordance with national guidelines and contribute to the delivery of shared, multidisciplinary, standardized, and longitudinal continuing education programs.

Adequate training is essential to prevent—or at least mitigate—the risk of automation bias, defined as the uncritical or excessive reliance of clinicians on automated decisions and recommendations [56].

SIMI is strongly convinced that clinicians will critically evaluate and effectively utilize AI tools in daily practice only if AI education will be integrated in all levels of medical training. Therefore, we recommend a reduced automation bias and improve the safe adoption of AI technologies, leading to better patient outcomes aligning with real-world clinical needs.

3. **AI technologies should be developed with the explicit goal of reducing administrative and cognitive workload for physicians and healthcare staff, thereby enhancing the quality and efficiency of patient care.**

**Table:** 

Possible risks	Weaknesses	Potential solutions
Privacy violations from using general-purpose AI tools	Lack of certified platforms for clinical use	Use certified, GDPR-compliant healthcare-specific platforms
Overdependence on automation for documentation	Clinicians may lack training in AI tools	Provide continuous education and maintain clinician oversight

The considerable administrative and cognitive burden placed on healthcare professionals must not be underestimated. Current healthcare work structures require substantial time, psychological effort, and emotional energy for tasks that are not directly related to patient care [57, 58]. Over time, there has been a marked increase in bureaucratic duties, which has reduced time available for direct patient interaction, communication with families, clinical reasoning, and the optimization of therapeutic strategies—factors contributing to both clinician and patient-related burnout [59].

The widespread adoption of Electronic Health Records (EHR), Picture Archiving and Communication Systems (PACS), and laboratory information systems—often integrated—has already simplified certain aspects of clinical work, particularly information retrieval and documentation, and contributed to the standardization of clinical processes [60].

Artificial Intelligence has the potential to further enhance these systems by streamlining cognitively demanding workflows and automating repetitive tasks [61]. These include the retrieval of relevant anamnestic data (especially from connected EHRs), correction of redundancies or errors in clinical documentation, assisted completion of clinical-administrative forms (such as hospital discharge summaries [SDOs], electronic prescriptions, or multidimensional assessment tools), and the application of diagnostic and prognostic scoring algorithms based on patient data [62, 63]. Currently, many clinicians independently use general-purpose generative AI tools for such purposes [14], and several studies indicate that generative AI can improve performance on patient-care tasks [64]. These virtual assistants can help uncover clinical scenarios that might otherwise be overlooked, while final judgment remains with the physician.

In addition, several domain-specific software tools are under development or evaluation, based on advanced Natural Language Processing (NLP) technologies. Examples include AutoScribe, Suki AI, and Ambience Healthcare, which are designed specifically to support clinical practice [65].

The primary challenge in this domain is the protection of personal health data, particularly with respect to compliance with the General Data Protection Regulation (GDPR). The use of personal health information in uncontrolled environments—such as third-party chatbot interfaces—raises the risk of privacy violations, including unauthorized access, accidental data disclosure, user profiling, biased AI-generated outputs with discriminatory potential, and re-identification of pseudonymized data [66].

For these reasons, in addition to continuous training and awareness initiatives—an area where SIMI should take a leading role—the implementation of AI technologies must occur within certified, healthcare-specific software platforms. This should be accompanied by the adoption of codes of conduct and certification frameworks by healthcare institutions to ensure legal and ethical compliance.

Adopting AI solutions to streamline administrative and cognitive tasks is expected to free up clinicians’ time, allowing greater focus on direct patient care. This recommendation will likely improve workflow efficiency and reduce burnout among healthcare staff, contributing to higher job satisfaction and better patient experiences, by automating repetitive processes. Therefore, we expect AI can help optimize resource allocation and support the delivery of high-quality, timely care.

4. **AI and other technological innovations should actively contribute to reducing—rather than perpetuating—existing disparities in health and access to care. To support this objective:**
● AI systems should be trained on data from diverse and representative settings and populations;
● Public institutions, including the Italian government and relevant health authorities, should invest in research aimed at identifying and mitigating any discriminatory outcomes associated with AI technologies;
● Multidisciplinary collaborations involving governmental bodies, academic institutions, nonprofit organizations, and private industry should be established to promote the development of fair and unbiased AI algorithms, both now and in the future.

**Table:** 

Possible risks	Weaknesses	Potential solutions
Algorithmic bias from non-representative datasets	Underrepresentation of minority populations in training data	Train AI on inclusive datasets and promote universal design principles
Emergence of a two-tiered healthcare system	High costs and lack of digital literacy in underserved areas	Public investment, accessible interfaces, and support personnel for vulnerable groups

Deep-rooted social inequalities persist within today’s healthcare systems, affecting both access to care and participation in basic and clinical research. In this context, AI systems present a dual potential: they may help mitigate these disparities or, conversely, exacerbate them.

Certain groups remain particularly vulnerable in terms of equitable access to healthcare. These include individuals from specific ethnic backgrounds impacted by large-scale migration, persons with disabilities, and those affected by rare diseases [67, 68]. To be effective in underserved populations, AI tools—whether general-purpose chatbots or CDSS—must be designed with these minority groups explicitly in mind. However, AI systems are often trained on standard datasets influenced by the priorities of commissioning entities, which frequently exclude or underrepresent these populations. This omission can overlook crucial genetic, epigenetic, and socio-environmental factors that affect disease development and treatment response.

As a result, algorithmic bias may arise, manifesting in generalization errors, misclassifications, inaccurate probability estimates, and flawed interpretations of clinical variables—ultimately leading to erroneous prognostic or therapeutic suggestions [69]. To address this, even correction strategies such as cross-validation, post hoc interpretability assessments, and internal checkpoints must be designed with underrepresented populations in mind. AI software developed using dedicated datasets that reflect the characteristics of minority populations may offer a path toward reducing disparities in both access and quality of care. Moreover, such efforts could contribute to generating new scientific knowledge that transcends current research limitations [70].

A related concern is the potential emergence of a two-tiered healthcare system. The costs associated with acquiring, updating, and maintaining AI systems—as well as training personnel—may be unsustainable for many healthcare institutions, particularly those in the public sector. As a result, AI systems calibrated for minority populations could become accessible only to wealthier regions or private entities, limiting availability for those most in need [71, 72]. Furthermore, even when available, these tools may be ineffective if patients lack the necessary education to understand their capabilities and risks. Cultural and material barriers to digital healthcare environments remain significant for many disadvantaged populations. Addressing this challenge requires dedicated personnel to support individuals—particularly in low-income communities—who face technological access issues [50].

In this regard, SIMI is committed to actively contributing during the design and validation phases of medical AI software, offering guidance to address the underrepresentation of vulnerable populations. It also aims to serve as an intermediary between ethics committees and developers. SIMI will promote the development of applications aligned with the principles of universal design, digital accessibility, and comprehensibility for users with cognitive disabilities. Moreover, SIMI will play a key role in clinician education—not only regarding the general principles of responsible AI use, but also in supporting access for minority populations. This includes helping patients with limited or no digital literacy benefit from AI-driven innovations.

This recommendation will help ensure that vulnerable and underserved populations benefit from AI-driven innovations, reducing disparities in access and outcomes. This is particularly important in limited sources scenarios, to support the best choose of diagnostic–therapeutic flowchart, also where medical equipment are scarce. Multidisciplinary collaboration and public investment in fair AI development will foster inclusivity and support the creation of universally accessible healthcare technologies.

5. **To foster public trust and uphold the integrity of the clinician–patient relationship, transparency throughout all stages of AI development and clinical application is needed. Whenever feasible, patients and healthcare providers should be informed when AI systems are involved in diagnostic or therapeutic processes.**

**Table:** 

Possible risks	Weaknesses	Potential solutions
Lack of disclosure may erode patient trust	Opaque AI decision-making processes	Inform patients and clinicians when AI is used; adopt explainable AI tools
Misalignment with clinical needs	Limited clinician involvement in development	Involve clinicians in AI design, development, and validation

AI must be thoughtfully integrated into clinical practice, and SIMI emphasizes the importance of explicitly disclosing its use in all procedures and clinical workflows where it is involved. At the core of healthcare lies the clinician–patient relationship, which is grounded in mutual trust. Transparency in clinical decision-making and treatment selection, including drug prescriptions, is essential to maintaining and reinforcing this trust. Conversely, a lack of transparency risks undermining it.

Human capabilities are inherently limited, and collaborative teamwork is often necessary to address individual constraints. In this context, AI can serve as a valuable ally, supporting healthcare teams in mitigating knowledge gaps. However, its use must be clearly acknowledged, akin to any other diagnostic or therapeutic tool.

Such transparency not only helps patients better understand the complexity and rigor of the diagnostic process but also allows clinicians to demonstrate the care and diligence underlying their clinical decisions—decisions increasingly supported by advanced technologies.

Furthermore, the development of AI-based tools must follow transparent and ethical standards. SIMI advocates for the adoption of explainable AI tools that produce clear, interpretable outputs which clinicians can understand and trust.

To ensure clinical relevance and safety, SIMI also recommends the active involvement of clinicians in development teams. Their expertise is crucial to aligning technological innovation with real-world clinical needs. In this regard, SIMI encourages developers to collaborate with established and recognized medical scientific societies or professional organizations throughout the design, development, and validation of AI technologies in healthcare.

By fostering transparency, collaboration, and ethical development, AI can serve as a powerful support system—enhancing, rather than replacing, the human elements that define high-quality care. This model of co-development is central to the vision of Symbiotic AI, in which human and AI are integrated within a mutually reinforcing partnership.

SIMI expects and encourages the transparency in the development and clinical application of AI. This process will strengthen public trust and uphold the integrity of the clinician–patient relationship. Informing patients and providers about the involvement of AI in care processes is expected to enhance understanding, acceptance, and shared decision-making. At the same time, the adoption of explainable AI tools will facilitate clinician oversight and accountability, supporting safer and more ethical integration of this technology in medical practice.

6. **The critical importance of safeguarding the privacy and confidentiality of both patient and clinician data during the development, training, and deployment of AI models in clinical practice should be emphasized.**

**Table:** 

Possible risks	Weaknesses	Potential solutions
Re-identification from anonymized data	Inadequate data governance frameworks	Implement strict access controls and traceability mechanisms
Misuse of patient data	Lack of infrastructure for secure hosting	Host data within national healthcare systems under privacy laws

The integration of AI into clinical practice requires the use of patient-care data for the development, training, and validation of AI models. While AI’s advanced capabilities in data processing and pattern recognition offer significant potential, they also heighten the risk of re-identification—even when datasets have been anonymized. Nonetheless, such data are essential for developing tools that effectively support clinical decision-making and improve patient outcomes. It is imperative that the use of AI does not undermine the trust that forms the foundation of the clinician–patient relationship.

Clinical data—whether relating to patients or the healthcare professionals involved in their care—are inherently reflective of the clinician–patient dynamic. The inappropriate use or absence of such data may compromise the continuity and quality of care. Given AI’s advanced analytic capabilities, the risk of re-identification from anonymized datasets remains a critical concern.

Accordingly, SIMI considers the protection of privacy a non-negotiable prerequisite for the ethical development and deployment of AI-based tools in healthcare. Both personal and clinical data must remain under the ownership and control of the patient and should be accessed and used by clinicians exclusively to enhance care, whether in routine clinical practice or ethically approved research [73, 74].

To support this principle, SIMI advocates for the establishment of robust data governance frameworks. These should include strict access controls, traceability of data usage, and full compliance with national and international data protection regulations. Where feasible, SIMI recommends that data repositories be hosted within the national healthcare system’s infrastructure, in accordance with applicable privacy laws and up-to-date, authoritative technical standards for clinical data use [73, 74].

The integration of AI into healthcare must be guided by the principles of transparency, accountability, and respect for patient autonomy. Protecting privacy is not only a legal obligation but also a foundational requirement for preserving the integrity of the physician–patient relationship in the digital era.

This recommendation should reduce the risk of data breaches and unauthorized use, ensuring compliance with legal and ethical standards. Emphasizing robust data privacy and confidentiality measures will protect patient and physician trust, which is essential for effective healthcare. This goal must be pursued by implementing rigorous governance frameworks that support the responsible use of clinical data, fostering innovation while protecting individual rights.

7. **Development, validation, and application of AI in healthcare must align with the core principles of medical ethics. These technologies should aim to enhance patient care, support clinical decision-making, strengthen the clinician–patient relationship, and promote fairness and equity within the healthcare system.**

**Table:** 

Possible risks	Weaknesses	Potential solutions
AI may prioritize efficiency over equity	Risk of replacing human judgment	Design AI to enhance fairness and support clinical autonomy
Reinforcement of structural inequities	Biased or narrowly scoped training data	Use inclusive datasets and ethical design principles

AI has the potential to enhance healthcare delivery by enabling faster and more accurate disease detection, greater personalization of treatments, and more efficient allocation of resources. However, these improvements must be guided not only by considerations of efficiency, but also by a clear ethical imperative: ensuring that every patient receives appropriate, timely, and evidence-based care. As emphasized by the World Health Organization (WHO, 2021), AI technologies should contribute to a more responsive model of medicine—one focused on the holistic well-being of patients and designed to reinforce, rather than replace, human intervention [50]. This objective should be pursued in accordance with the bioethical principles outlined by Tom L. Beauchamp and James F. Childress (1979), which are already embedded in clinical practice, and further strengthened by the principles of explicability and responsibility [75].

The enhancement of clinical decision-making is a critical area in which AI can complement clinicians’ professional expertise [42, 44, 45]. Predictive algorithms, diagnostic imaging analysis, and decision support systems should be designed to assist—rather than replace—clinical judgment, enriching the decision-making process with additional data while safeguarding the autonomy and accountability of healthcare professionals. In the context of Internal Medicine, AI should aim not only to advance technological capabilities but also to promote health equity and genuinely person-centered care.

AI technologies also offer the potential to reduce administrative and analytical burdens. However, as highlighted in the WHO’s ethical guidelines, technologies trained on biased or narrowly scoped data may reinforce existing structural inequities. AI must serve as a tool for promoting fairness and justice within healthcare systems. To this end, algorithmic models must be developed using inclusive and representative datasets, to mitigate the risk of perpetuating or exacerbating disparities based on gender, ethnicity, socioeconomic status, or geographic location. Moreover, well-designed AI systems can help bridge care gaps by improving access in rural and underserved areas.

According to this recommendation, SIMI expects to support clinical autonomy and accountability, preventing the replacement of human judgment by automated systems. The use of inclusive datasets and ethical design principles will help mitigate structural inequities and foster a more just healthcare system. Aligning AI development and application with core medical ethics will ensure that technological advances genuinely enhance patient care and promote fairness.

8. **Clinical safety, effectiveness, and equitable use of AI technologies should be prioritized by all stakeholders involved—including developers, researchers, implementers, and regulators. The integration of AI into healthcare should follow a process of “continuous improvement”, incorporating real-world testing, feedback from end users, and rigorous scientific validation across diverse clinical settings. Particular consideration must be given to the identification and management of both current and emerging risks associated with AI use in medicine.**

**Table:** 

Possible risks	Weaknesses	Potential solutions
Static AI systems may become outdated	Lack of real-world validation and user feedback	Adopt continuous improvement and multicenter clinical trials
Regulatory gaps for evolving AI systems	Inflexible approval models	Update frameworks to accommodate dynamic AI systems and human oversight

Any AI system intended for medical use should not be conceived as a static product but rather as a dynamic system subject to ongoing refinement. This necessitates a process of continuous improvement, which includes not only controlled preliminary testing but, more importantly, rigorous scientific validation in real-world clinical settings that are diverse and complex. Such validation must actively involve all end users—physicians, other healthcare professionals, and patients—whose feedback is a critical resource for fine-tuning and adapting algorithms to the practical demands of everyday medical practice.

In particular, AI solutions must undergo prospective, multi-center clinical trials to verify their safety and clinical utility in environments that differ from those in which they were originally developed. The current regulatory framework—predicated on the approval of products following successful clinical trials and subsequent market authorization—must be updated to accommodate the concept of “evolving” or “dynamic” systems. Future regulatory models should address the implications of updates and modifications made to AI systems after initial approval.

The concept of “human warranty,” as articulated in the WHO report, underscores the need for human oversight throughout every phase of AI implementation [50]. This ensures that clinical responsibility remains clearly attributable and traceable at all times.

This approach is expected to facilitate early identification and management of emerging risks, supporting the sustainable integration of AI in healthcare. Prioritizing continuous improvement and real-world validation of AI technologies will ensure their safety, effectiveness, and adaptability across diverse clinical settings. In this regard, SIMI expects that engaging end users in feedback and validation processes will enhance the clinical relevance and usability of AI tools, ultimately improving patient outcomes.

9. **Developers should be held accountable not only for violations of binding legal obligations, but also for systematically disregarding recognized industry standards and ethical guidelines during the design and development of AI systems, especially where such standards contribute to risk mitigation and safe deployment. However, this does not exempt deployers (that is physicians and other health operators under their own authority) from their own responsibilities: these remain accountable for using AI systems in accordance with the manufacturer’s instructions, complying with applicable use conditions, and adhering to good operational practices, as explicitly required by the AI Act.**

**Table:** 

Possible risks	Weaknesses	Potential solutions
Unclear liability in case of harm	Developers may ignore ethical standards	Follow EU AI Act and Liability Directive for shared responsibility
Clinicians misusing AI tools	Lack of formal reporting mechanisms	Train deployers and implement adverse event reporting systems

While SIMI’s position on developer accountability reflects important ethical considerations, the current European regulatory landscape introduces a more nuanced framework that warrants careful examination. The EU AI Act (Regulation 2024/1689) and the forthcoming AI Liability Directive (Directive 2024/2853) establish a graduated and shared liability model, rather than assigning automatic responsibility to developers for the outcomes of AI systems. This framework is grounded in several key principles: the proportionality of responsibility relative to the degree of control exercised over the system, the risk classification of the AI application (invariably “high-risk” in medical contexts), and the respective roles of providers and deployers across the development and deployment lifecycle [76, 77].

The regulatory architecture acknowledges that accountability should correspond to actual influence and oversight capacity. Providers of high-risk AI systems are required to comply with a set of stringent obligations, including risk management protocols, robust data governance, detailed technical documentation, and—critically—post-market surveillance. In cases where providers fail to meet these standards or breach transparency obligations, the AI Liability Directive introduces a presumption of causality in favor of injured parties, thereby facilitating legal redress and reinforcing accountability.

However, this model of shared responsibility also makes clear that deployers—such as physicians and healthcare institutions—bear distinct and non-transferable duties. These include adherence to the intended use of the system, compliance with operational conditions, and the implementation of appropriate clinical oversight mechanisms. Rather than creating parallel tracks of accountability, the European framework establishes an integrated system in which responsibility is distributed based on actual capacity to prevent harm and ensure the safe deployment of AI tools. This approach reflects the complex, multi-stakeholder nature of AI implementation in healthcare, while maintaining clearly defined standards for both technological development and clinical practice.

In addition, we advocate for the implementation of formal procedures for reporting and investigating adverse events, near misses, and other safety-related incidents linked to the use of AI in healthcare settings.

SIMI strongly expects that establishing clear accountability for both developers and users of AI systems will promote responsible innovation and safe clinical implementation. Therefore, this recommendation is expected to clarify legal responsibilities, reduce liability uncertainty, and encourage adherence to recognized standards and guidelines. The implementation of formal reporting and monitoring mechanisms will support ongoing risk mitigation and foster a culture of safety in AI-based healthcare.

10. ** It is of greatest importance to evaluate the environmental footprint of AI technologies. The sustainability and ecological impact of AI should be carefully assessed and mitigated across the entire lifecycle, from development through deployment.**

**Table:** 

Possible risks	Weaknesses	Potential solutions
High energy consumption of large models	No certification framework for environmental impact	Use sustainable methods like transfer learning and model optimization
Exacerbation of economic disparities	Lack of moderation in AI deployment	Limit AI use to clinically justified, high-value applications

Large-scale data and processing centers are being developed globally through both public and private initiatives, accompanied by the training of increasingly large and energy-intensive AI models. The economic and environmental costs associated with these technologies must not be overlooked [78]. We advocate for their development and adoption in ways that are both affordable and climate-conscious. The integration of AI into healthcare should not exacerbate existing economic disparities in access to care or compromise the environmental sustainability of the healthcare sector [79, 80].

To date, and to the best of our knowledge, there is no established certification or evaluation framework for assessing the environmental footprint of AI applications in healthcare, even as the broader impact of environmental factors across the medical lifecycle is beginning to receive attention [81]. As physicians, we are acutely aware of the rising costs of healthcare, the long-term sustainability challenges facing our health systems, and the increasing burden of morbidity and mortality linked to climate change [82]. Notably, the healthcare industry itself is one of the largest contributors to pollution worldwide [83].

Whenever feasible, AI development should prioritize existing approaches that enhance sustainability—such as transfer learning and domain adaptation—while efforts should be made to reduce the size and energy requirements of models without compromising their performance [84–87].

Until the environmental impact of AI technologies is meaningfully reduced, SIMI urges its members—and all healthcare stakeholders—to adopt these tools with discretion and moderation. Their use should be limited to well-justified, clinically appropriate purposes that demonstrably support the maintenance or continuous improvement of high-quality healthcare services, processes, and outcomes.

**Fig. 3:**
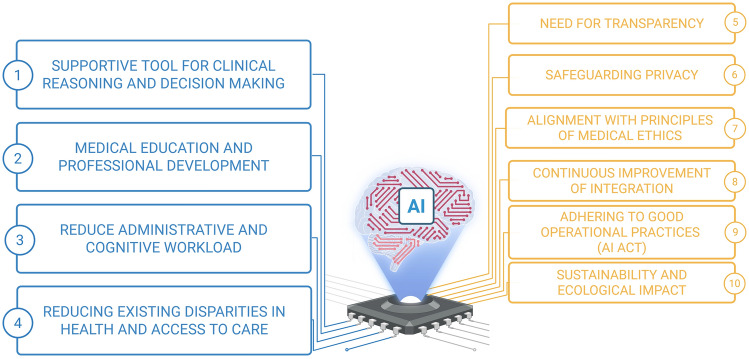
Summary of the SIMI position statements on Artificial Intelligence on the main opportunities (blue boxes) and concerns (yellow boxes) it may stimulate in clinical practice. The relative statement with commentary is indicated in the number included in the circle on the box external side. Created in BioRender. Cicco, S. (2025) https://BioRender.com/sibqraa

With this recommendation, we expect to encourage the adoption of energy-efficient methods and limit the use of resource-intensive models to clinically justified applications. By prioritizing environmental responsibility, healthcare stakeholders can help reduce the sector’s carbon footprint and support global efforts to combat climate change. This goal must be pursued by assessing and mitigating the environmental impact of AI technologies to support the long-term sustainability of healthcare systems (see Fig. 3).

Box 2: Traditionally, AI in medicine has followed a model-centric paradigm, where the primary focus is on developing increasingly sophisticated algorithms to achieve high predictive accuracy. In this approach, the training data is often treated as static and receives limited attention, while the model undergoes continuous refinement. Although this strategy can yield strong results in controlled research environments, it may falter in real-world clinical settings, where data is frequently noisy, incomplete, or biased. Neglecting data quality in such contexts can compromise the reliability, fairness, and safety of AI systems.

The data-centric AI paradigm addresses these limitations by shifting the emphasis from model optimization to data improvement. Here, the model architecture is typically held constant, while systematic efforts are made to enhance the quality of the data through cleaning, annotation, balancing, and ongoing curation. For instance, improving the consistency of radiology labels or resolving missing values in electronic health records can often yield greater gains in model performance and generalizability than further tuning of model parameters [88].

## Conclusion

The introduction of AI in healthcare not only promises to improve the efficiency and accuracy of diagnoses and treatments but also offers the opportunity to further humanize healthcare. As repetitive tasks are automated, humans will be able to focus on tasks that are uniquely human: building relationships, exercising empathy, and using human judgment to guide and advise. AI can free healthcare professionals from administrative and repetitive tasks, allowing them to dedicate more time and attention to patients. This can lead to greater satisfaction for both patients and doctors, improving the quality of care and strengthening the doctor-patient relationship [88]. In addition, AI can help reduce medical errors, improve the management of chronic diseases, and optimize the use of healthcare resources. However, ethical and legal challenges should be addressed to ensure transparency in decision-making processes and build user trust. Continuous training of healthcare professionals on the use of AI and the adoption of rigorous standards for data protection are essential for the effective and safe integration of AI into medical practice.

Ultimately, AI has the potential to transform the healthcare sector, making it more efficient, precise, and human. With a careful and responsible approach, we can leverage technological innovations to improve people’s health and well-being, creating a future where technology and humanity work together for the common good.
